# Antiretroviral therapy suppressed participants with low CD4^+^ T-cell counts segregate according to opposite immunological phenotypes

**DOI:** 10.1097/QAD.0000000000001205

**Published:** 2016-09-07

**Authors:** Josué Pérez-Santiago, Dan Ouchi, Victor Urrea, Jorge Carrillo, Cecilia Cabrera, Jordi Villà-Freixa, Jordi Puig, Roger Paredes, Eugènia Negredo, Bonaventura Clotet, Marta Massanella, Julià Blanco

**Affiliations:** a University of California San Diego, La Jolla, California, USA; b Institut de Recerca de la Sida IrsiCaixa-HIVACAT, Institut d’Investigació en Ciències de la Salut Germans Trias i Pujol, UAB, Badalona; c Universitat de Vic – Universitat Central de Catalunya, Barcelona; d Fundació Lluita contra la SIDA, Institut d’Investigació en Ciències de la Salut Germans Trias i Pujol, Badalona, Spain; e Department of Microbiology, Infectiology and Immunology, Université de Montréal, Faculté de Médecine; f Centre de Recherche du CHUM, Montréal, Québec, Canada.

**Keywords:** cell death, discordant patients, immune activation, immune nonresponders, random forest, recent thymic emigrants

## Abstract

**Background:**

The failure to increase CD4^+^ T-cell counts in some antiretroviral therapy suppressed participants (immunodiscordance) has been related to perturbed CD4^+^ T-cell homeostasis and impacts clinical evolution.

**Methods:**

We evaluated different definitions of immunodiscordance based on CD4^+^ T-cell counts (cutoff) or CD4^+^ T-cell increases from nadir value (ΔCD4) using supervised random forest classification of 74 immunological and clinical variables from 196 antiretroviral therapy suppressed individuals. Unsupervised clustering was performed using relevant variables identified in the supervised approach from 191 individuals.

**Results:**

Cutoff definition of CD4^+^ cell count 400 cells/μl performed better than any other definition in segregating immunoconcordant and immunodiscordant individuals (85% accuracy), using markers of activation, nadir and death of CD4^+^ T cells. Unsupervised clustering of relevant variables using this definition revealed large heterogeneity between immunodiscordant individuals and segregated participants into three distinct subgroups with distinct production, programmed cell-death protein-1 (PD-1) expression, activation and death of T cells. Surprisingly, a nonnegligible number of immunodiscordant participants (22%) showed high frequency of recent thymic emigrants and low CD4^+^ T-cell activation and death, very similar to immunoconcordant participants. Notably, human leukocyte antigen - antigen D related (HLA-DR) PD-1 and CD45RA expression in CD4^+^ T cells allowed reproducing subgroup segregation (81.4% accuracy). Despite sharp immunological differences, similar and persistently low CD4^+^ values were maintained in these participants over time.

**Conclusion:**

A cutoff value of CD4^+^ T-cell count 400 cells/μl classified better immunodiscordant and immunoconcordant individuals than any ΔCD4 classification. Immunodiscordance may present several, even opposite, immunological patterns that are identified by a simple immunological follow-up. Subgroup classification may help clinicians to delineate diverse approaches that may be needed to boost CD4^+^ T-cell recovery.

## Abstract 2

Supplemental Digital Content is available in the text

## Introduction

Antiretroviral therapy (ART) leads to a rapid and sustained suppression of HIV-1 replication that allows for a reduction of T-cell activation and associated cell death favoring CD4^+^ T-cell recovery [1]. Despite this, some HIV-treated participants have a paradoxical response to ART, showing low increases of CD4^+^ T cells despite complete viral suppression [2,3]. These individuals are referred to as immunological nonresponders or immunodiscordant participants. In contrast, immunological responders or immunoconcordant individuals are able to recover CD4^+^ T-cell levels, even if ART is started with low CD4^+^ T-cell counts [4,5]. The suboptimal CD4^+^ T-cell recovery is a clinical concern as immunodiscordant participants show higher mortality rates and increased risk of clinical progression to AIDS-related and non-AIDS-related illnesses [6–11]. Several strategies have attempted to heighten CD4^+^ T-cell recovery in immunodiscordant participants; however, ART intensification with new antiretrovirals or complementation with immunomodulatory drugs has shown limited effects in increasing CD4^+^ T-cell counts [12–15].

Despite the clinical relevance of immunodiscordance, the lack of a consensus definition hampers the identification of immunodiscordant individuals, their clinical follow-up and ultimately the assessment of prevalence of immunodiscordance among HIV-treated individuals [16,17]. Under restrictive definitions, an absolute CD4^+^ T-cell count under 200 cells/μl after suppressive ART is considered an immunodiscordant response, although higher cutoff values of 350 and 500 cells/μl are also commonly used in the literature [16,17], as immune restoration above 500 cells/μl is associated with morbidity and mortality rates comparable with the ones observed in uninfected individuals [7]. An additional criterion for immunodiscordance considers CD4^+^ T-cell increases from ART initiation.

In the absence of standard definitions, multiple analyses have explored the immunological basis for immunodiscordance, that are strongly related to CD4^+^ T-cell homeostasis, in particular with poor thymic output, high cell turnover and sensitivity to ex-vivo cell death [16,17]. We considered that immunological features could be helpful to define a cutoff value for CD4^+^ T-cell recovery that in turn would help both to quantify the clinical impact of immunodiscordance and to explore new clinical interventions to reverse it. We approached this issue by using supervised random forest classification, a powerful machine learning algorithm characterized by high classification accuracy and the capacity to rank variables according to their importance for classification [18]. Our data suggest that CD4^+^ T-cell count of 400 cells/μl is the best immunological cutoff for classification. Importantly, after variable selection, unsupervised hierarchical clustering showed that immunodiscordant participants could be subgrouped according to three different immunological patterns that will probably require specific clinical interventions to reverse immunodiscordance.

## Materials and methods

### Individuals

We analyzed immunological data from 196 participants on suppressive ART (HIV RNA levels <50 copies/ml) for at least 2 years. All participants having the full immunological dataset were selected from a larger previous cross-sectional, descriptive and comparative study (*n* = 230) performed to analyze the immune status of participants with paradoxical immune response [19,20]. The Institutional Review Board approved the study (EO code: EO-07-024). Written informed consent was obtained from all participants before study enrollment. Clinical and demographic data for cross-sectional samples and for longitudinal analyses of CD4^+^ and viral load (VL) were collected from medical records.

### Immunological datasets

Most of collected data were generated in previous studies [19–22]. Previously reported immunological data were obtained using the following antibody combinations: CD45RA-FITC, CD31-PE, CD38-PerC-Cy5.5, CD3-APC-Cy7, CD4^+^-APC and CD8^+^-PE-Cy7; CD95/FAS-FITC, PD-1-PE, human leukocyte antigen - antigen D related (HLA-DR)-PerCP-Cy5.5, CD3-APC-Cy7, CD4^+^-APC and CD8^+^-PE-Cy7; and HLA-DR-FITC, CD38-PerCP-Cy5.5, CD45RO-APC, CD3-APC-Cy7 and CD8^+^-PE-Cy7. CD4^+^ and CD8^+^ T-cell death data from fresh peripheral blood mononuclear cells and data from soluble CD14 in plasma samples had been also previously reported [19,21]. A total of 57 immunological and cell-death parameters were collected for each participant (Supplementary Table 1). Unpublished data on cytomegalovirus (CMV)-specific IgG and IgM antibodies were measured using the semiquantitative BIO-FLASH CMV IgG and the qualitative BIO-FLASH CMV IgM chemiluminescent immunoassays, respectively (Biokit, Barcelona, Spain). Additional immunological parameters were available from a subset of participants of the initial cohort (*n* = 50) ([22] and unpublished data). Antibody combination were designed for regulatory T cells (Treg) and cell proliferation by flow cytometry as follows: Ki67-FITC, FOXP3-PE, CD25-PE-Cy7, CD5-PerC-Cy5.5, CD127-Alexa Fluor 647, CD3-APC-Cy7, CD4-V450 and CD8-V500; and T-cell maturation and immunosenescence as follows: CD3-APC-Cy7, CD4-PerCP-Cy5.5, CD8-V500, CD57-FITC, CD27-APC, CD28-PE, CCR7-PE-Cy7 and CD45RA-V450. A further collection of 48 parameters was available for this subset of participants (Supplementary Table 1).

### Statistical analysis

Participants were grouped as immunodiscordant or immunoconcordant based on either the CD4^+^ T-cell counts at the time of visit (using different cutoff values ranging from 200 to 600 cells/μl) or based on the increase in CD4^+^ cell counts from recorded CD4^+^ nadir values to CD4^+^ T-cell counts at the time of visit (ΔCD4, using different values ranging from 50 to 500 cells/μl). Participants below or above the indicated values were considered as discordant (unfavorable immunologic response) or concordant (favorable immunologic response), respectively.

Supervised random forest classification was performed using all sociodemographical, clinical and immunological variables collected as predictors using R Statistical Software (Free Software Foundation) [23] using the package randomForest [24]. Validation, classification performance and overfitting assessments were evaluated using a 10-fold cross-validation. The importance of predictors for classification was measured using the mean decrease in Gini index. Cutoff and ΔCD4 classificatory values were selected according to the classification accuracy evaluated by the combination of sensibility and specificity and the unbiased estimation of the misclassification error by the OOB (out-of-bag measure) error rate provided by random forest.

Using the variables with the highest contribution to sample discrimination (according to Gini's index), unsupervised hierarchical clustering in conjunction with heatmaps were performed to find patterns in our data in an unbiased fashion. Each variable was centered with respect to the mean and scaled by the SD. The distance between samples and variables was measured by means of Euclidean distance, and hierarchical clustering was performed using the Ward method. Principal component analysis (PCA) was performed to visualize the behavior of the cluster groups regarding different cellular processes.

Continuous variables were expressed as the median (interquartile range) and compared using Kruskal–Wallis (for multiple comparisons), Mann–Whitney *U* with permutation (for unbalanced groups) or signed-rank test (for paired analyses). Discrete variables were described as percentages and compared using the Fisher's exact test. Multiple comparisons were adjusted for false discovery rate. Statistical analyses were also performed using R Software version 3.0.2 [23] with two-tailed significance levels of 5%.

## Results

### Participant characteristics

Participants included in this analysis have been previously characterized [19,20]. The initial cohort contained 230 virologically suppressed HIV-infected individuals with a wide range of CD4^+^ T-cell recovery defined by CD4^+^ T-cell counts at the sampling time. However, the full set of immunological parameters for random forest analysis was available for 196 participants (Supplementary Fig. 1). The main characteristics of the analyzed cohort are shown in Table 1. Different CD4^+^ T-cell count strata showed similar sex representation, ART composition, hepatitis C virus (HCV) or hepatitis B virus (HBV) coinfection, time from diagnosis and time on ART; however, participants with poorer CD4^+^ T-cell recovery tended to be older (*P* = 0.02, one-way Kruskal–Wallis test). In addition, differences in CD4^+^ T-cell counts and CD4/CD8 ratio, individuals with poorer CD4^+^ T-cell recovery showed lower nadir CD4^+^ T-cell count values and lower CD8^+^ T-cell counts (*P* < 0.0001 in both cases, one-way Kruskal–Wallis test).

A wider range of immunological data, including Treg (CD25^+^FOXP3^+^ cells), proliferation (Ki67 expression), differentiation and immunosenescence [22] were available for 50 participants of the original cohort. The CD4^+^ T-cell stratification of this subset of individuals is also shown in Supplementary Fig. 1.

### Analysis of CD4^+^ cutoff values and ΔCD4^+^ as classifiers for immune recovery

**Fig. 1:**
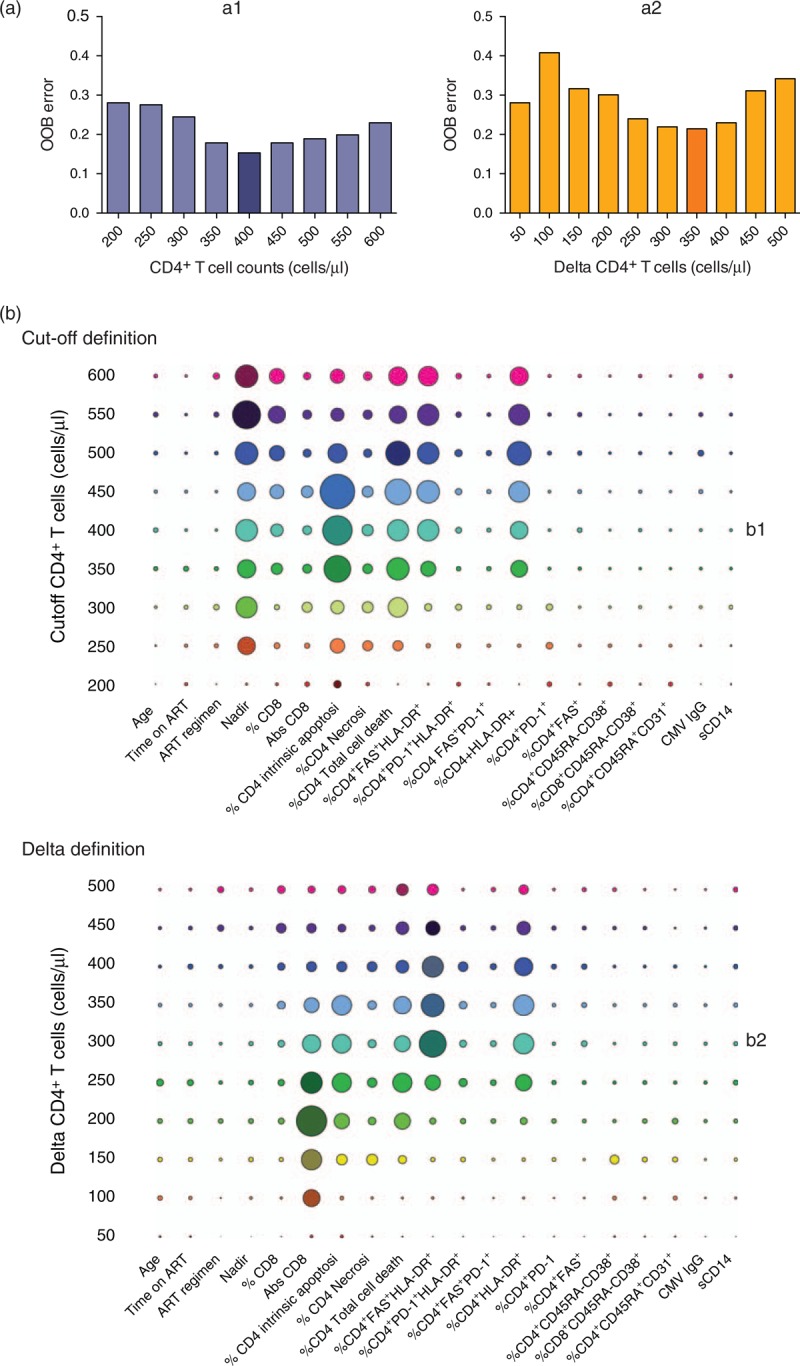
Comparison of CD4^+^ T-cell counts and increase cutoff values as classifiers for immune recovery.

To define the main immunological features that distinguish immunodiscordant and immunoconcordant participants, we analyzed the performance of different definitions of immunodiscordance using a random forest approach in which 74 immunological and clinical parameters were analyzed (Supplementary Table 1). Definitions were based on the CD4^+^ T-cell counts by establishing different cutoffs of immunodiscordance (CD4^+^ T-cell counts from 200 to 600 cells/μl) or based on the increase of CD4^+^ T-cell counts from nadir values (ΔCD4 values were set from 50 to 500 cells/μl). For cutoff values, the best classification was achieved using a value of 400 cells/μl that provided a specificity of 83% and a sensitivity of 88%, resulting in an OOB error of 15%. For ΔCD4 values, the best classifier was 350 cells/μl; although this classification showed a 84% of specificity, the sensitivity was significantly lower (76%), resulting in a OOB error of 20%, failing to achieve better classification than the cutoff values (Fig. 1a). Area under the curve (AUC) values confirmed these analyses and showed better classification for CD4^+^ T-cell count cutoff value 400 cells/μl (AUC 90%) than for ΔCD4 value 350 cells/μl (AUC 84%).

(a) The performance of different definitions of immune recovery according to CD4^+^ T-cell counts (a1) or CD4^+^ T-cell increases (ΔCD4, a2) was assessed by analyzing the out-of-bag error. The best classifier in each case is highlighted. (b) Summary of variables of importance for each classification (panel b1 cutoff values, panel b2 increase in CD4^+^ T-cell count values). For each classifier, the size of the symbol indicates the Gini index. The highest Gini index for each classification is highlighted. Abs, absolute counts; CMV, cytomegalovirus.

The analysis of relevant variables that might contribute to the immunodiscordant phenotype in each definition is shown in Fig. 1b. These results highlight different immune characteristics depending on the threshold of CD4^+^ T-cell counts analyzed. Restrictive definition of discordance (cutoffs CD4^+^ T-cell count 200 cells/μl) showed relevant contributions of nadir, recent thymic emigrants (RTE, CD4^+^CD45RA^+^CD31^+^) and PD-1 expression, activation (CD38) and cell death in CD4^+^ T cells to immunodiscordance. However, at higher cutoff values, the parameters related to activation (HLA-DR expression) and death of CD4^+^ T cells as well as nadir increased their relevance for classification. Activation and death of CD4^+^ T cells also showed importance for classification in ΔCD4 classification, although the absolute CD8^+^ T-cell count was unexpectedly relevant for lower and intermediate ΔCD4 values, and the coexpression of CD95 and HLA-DR in CD4^+^ T cells were highly relevant for ΔCD4 values above 250 cells/μl. In summary, immunological parameters seem to better classify participants according to the value of circulating CD4^+^ T cells achieved, rather than to the global increase of CD4^+^ T-cell counts.

### Unsupervised clustering of data

**Fig. 2:**
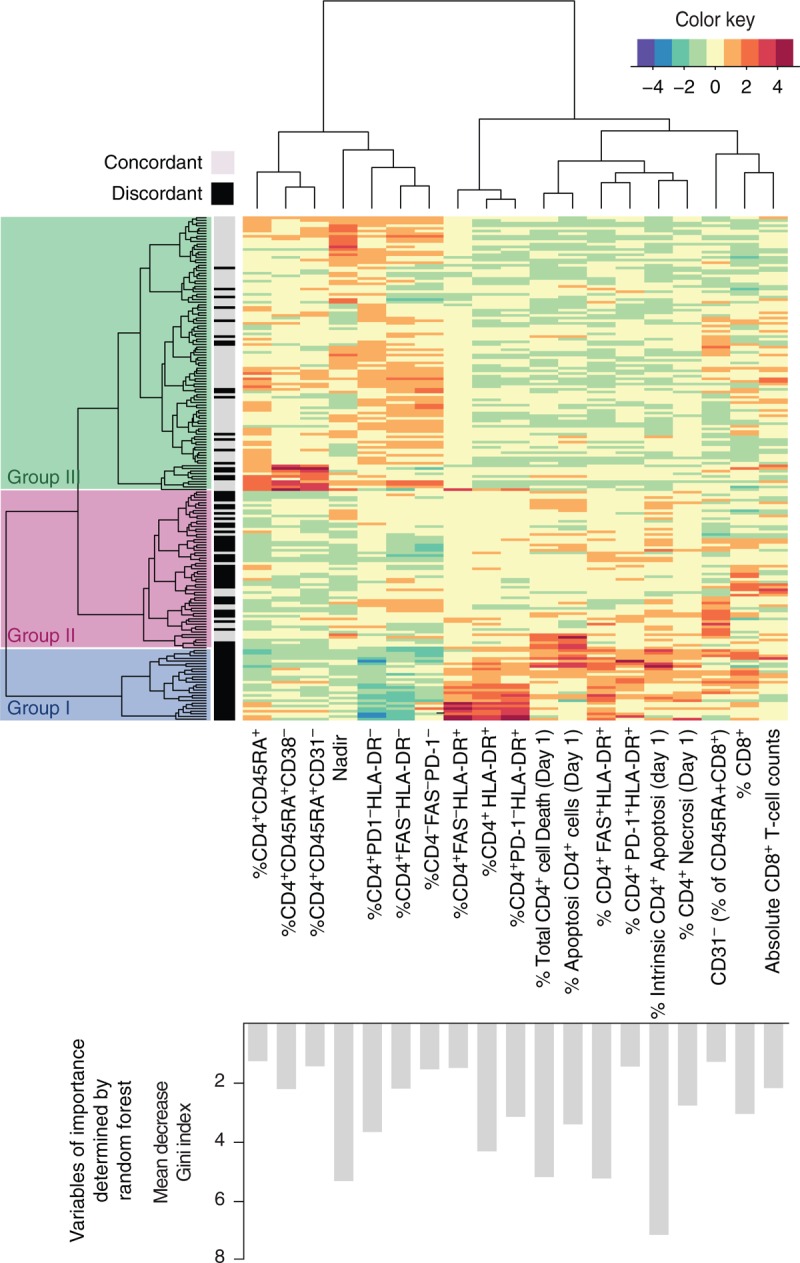
Clustering of participants according to variables of importance of the CD4^+^ T-cell count cutoff 400 cells/μl.

Variables of importance obtained from the optimal cutoff value (CD4^+^ T-cell count 400 cells/μl) random forest approach (highest Gini score) were used in an unsupervised hierarchical clustering analysis (Fig. 2). Two main clusters were clearly identified: a smaller one (Group I) containing exclusively immunodiscordant participants with high activation and death of CD4^+^ T cells and a low naive (CD45RA^+^) CD4^+^ T cells, and a larger cluster including all concordant individuals with intermingled immunodiscordant participants. This cluster can be subdivided in two groups, one of them (Group II) encompassing mostly immunodiscordant participants and the other one (Group III) that included mostly immunoconcordant individuals. Main differences between these latter groups were lower naïve CD4^+^ T cells, higher activation and death of CD4^+^ T cells in Group II compared with Group III. A similar cluster structure was obtained using parameters of importance for the ΔCD4 350 cells/μl classification (Supplementary Fig. 2).

Heatmap of 191 individuals generated from activation, cell death and RTE frequency data shows one main group of immunodiscordant individuals (Group I) and a major group of intermingled immunodiscordant and immunoconcordant individuals that has been subdivided in Group II and Group III. Color key is shown in the upper right side, and Gini index for each variable is shown in the lower histogram.

We further explored whether information on T-cell differentiation, proliferation and Treg frequency (Supplementary Fig. 2 and Table S1) could modify the classification of immunodiscordant individuals. The analysis of variables of importance and the resulting heatmap for the cutoff CD4^+^ T-cell count 400 cells/μl show again a relevant weight of activation and proliferation markers (Supplementary Fig. 3), with a low segregation performance for the frequency of naïve cells or the differentiation status of T cells. In contrast, the frequency of Tregs and specifically the proliferation of this subset appears as a major contributor to segregation.

### Immunological patterns of clustered individuals

**Fig. 3:**
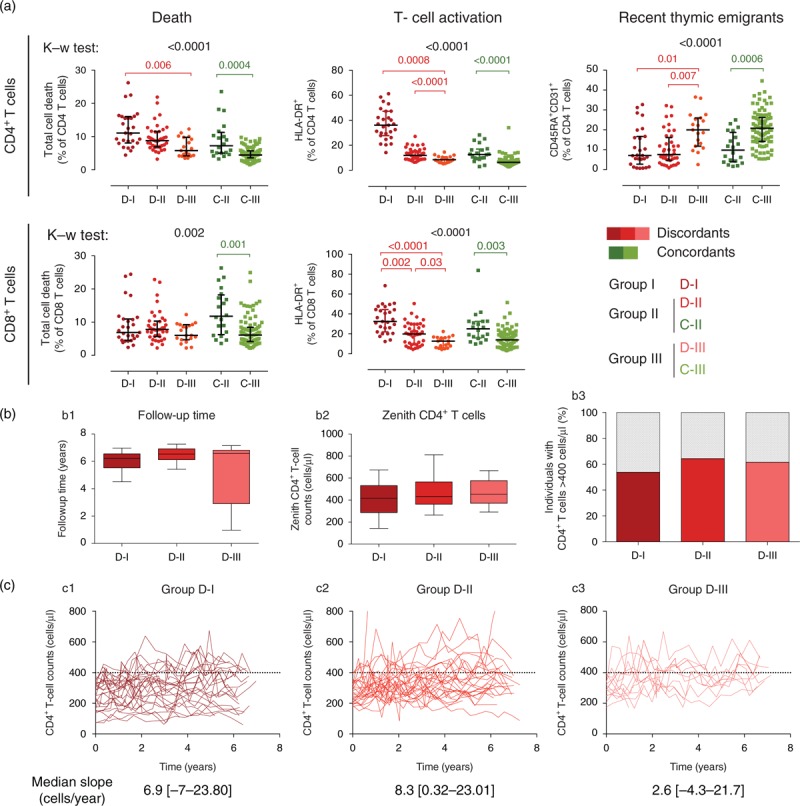
Analysis of clustered individuals.

To evaluate the immunological and clinical differences among clustered individuals, we defined and compared immunoconcordant and immunodiscordant groups in each cluster (Fig. 2). Data summarized in Table 2 and Fig. 3a show that immunodiscordant individuals clustered in Group I, II and III (D-I, D-II and D-III, respectively) and immunoconcordant individuals clustered in Groups II and III (C-II and C-III, respectively) showed similar time of infection, time on ART, ART composition (protease inhibitor vs non-nucleoside reverse transcriptase inhibitors) and coinfections (HVB, HCV). However, differences were observed in age and sex. Immunoconcordant tended to be younger than immunodiscordant (irrespective of the cluster), whereas the frequency of women was higher in Group III, and no women were found in Group I. Importantly, no differences in nadir, absolute CD4^+^ T-cell counts or CD4^+^ T-cells increases were observed among immunoconcordant subgroups. A detailed analysis of immunological features of each subset confirmed previous data associated with poor CD4^+^ T-cell recovery but also revealed surprising features in some groups. Among immunoconcordant participants, Group C-II showed lower CD4/CD8 ratio, lower RTE frequency but higher activation in both CD4^+^ and CD8^+^ T cells compared with Group C-III. Among immunodiscordant groups, Group D-I showed the expected profile with lowest RTE frequency, highest levels of CD4^+^ T-cell activation (HLA-DR^+^), PD-1 expression and cell death, both caspase-dependent or caspase-independent (Fig. 3a and Table 2). Cluster D-III showed an unexpected high RTE frequency with low values of CD4^+^ T-cell activation, PD-1 expression and death, although the latter parameters were still significantly higher when compared with immunoconcordant participants clustered in Group C-III (Fig. 3a and Table 2). Importantly, cluster D-III showed additional specific features: low platelecrit, leukocyte and lymphocyte count, low CD8^+^ T-cell count and low levels of activation (as measured by HLA-DR expression, Supplementary Fig. 4). Immunodiscordant individuals clustered in Group D-II showed an intermediate pattern and were characterized by low RTE frequency, intermediate cell death and a relatively low activation in the CD4^+^ T-cell compartment (Fig. 3 and Table 2). The full set of variables was analyzed in a PCA approach showing that, although irrelevant for immunodiscordant identification, CD8^+^ T-cell variables also contribute to subgrouping of participants (Supplementary Fig. 5). Importantly, from a clinical point of view, a simple combination of three parameters measuring CD45RA, HLA-DR and PD-1 expression in CD4^+^ T cells is able to classify immunodiscordant participants in Groups D-I, D-II and D-III with an overall 81.4% accuracy, suggesting that a much simpler strategy using a single panel of flow cytometry staining could be easily implemented in the routine clinical follow-up (Supplementary Fig. 6). In summary, our data provide immunological evidence for a subclassification of HIV-infected individuals (both immunodiscordant and immunoconcordant) and underscores the existence of a subset of immunodiscordant individuals with a paradoxical immune profile comparable with immunoconcordant (low activation and cell death, and high RTE frequency), suggesting that the immunodiscordant phenotype defined by CD4^+^ T-cell counts below 400 cells/μl may accommodate very different, even opposite immunological profiles.

(a) Immunodiscordant individuals clustered in Groups I, II and III (D-I, D-II and D-III, dark, intermediate and light red symbols, respectively) and immunoconcordant individuals clustered in Groups II and III (C-II and C-III, dark and light green symbols, respectively) were analyzed for the indicated immunological parameters. The five groups were compared statistically using the Kruskal–Wallis test (*P* values indicated in black) and differences within immunodiscordant (*P* values in red) and immunoconcordant (*P* values in green) are shown in each graph. Panels (b) and (c) show the analysis of CD4^+^ T-cell count evolution in immunodiscordant subgroups. (b) The follow-up of different individuals (b1), the maximal CD4^+^ T-cell counts achieved by each individual (b2) and the percentage of individuals achieving CD4^+^ T-cell count more than 400 cells/μl is shown for each immunodiscordant subgroup. No significant differences were observed. (c) Spaghetti plots of CD4^+^ T-cell count evolution for each individuals in immunodiscordant subgroups I (c1), II (c2) and III (c3). *X* axis indicates time from inclusion into the cohort; ART was initiated at least 2 years prior to inclusion. Black dotted line indicates the 400 cells/μl threshold that defines immunodiscordance. The median slope and interquartile range for each subgroup is shown. No differences were observed between subgroups.

### Evolution of CD4^+^ T-cell counts in immunodiscordant subgroups

To address the clinical evolution of the different subgroups of participants, we collected CD4^+^ T-cell counts and VL data from sampling time (2007–2008) to April 2015. Follow-up criteria were similar to inclusion criteria; individuals analyzed showed undetectable VL (one blip <500 copies/ml was allowed) and did not receive pegylated Interferon (PEG-IFN) or chemotherapy treatment. Data were available for 67 immunodiscordant individuals (26 from subgroup D-I, 28 from D-II and 13 from D-III). Collected data showed a median follow-up time of 6.4 years and were similar for all three subgroups (Fig. 3b). Surprisingly, no significant differences could be detected among immunodiscordant subgroups in the CD4^+^ zenith or the percentage of participants reaching CD4^+^ T-cell counts greater than 400 cells/μl over the follow-up period (Fig. 3b). Furthermore, the slope of CD4^+^ T-cell count over the follow-up period was similar for all three groups (Fig. 3c), with a median increase (slope) of CD4^+^ T cells during the period of 6.9 (−7 to 23.8), 8.3 (0.3–23.0) and 2.6 (−4.3 to 21.7) cells/year for Groups D-I, D-II and D-III, respectively. Taking all together, our data demonstrate that a similar blunted CD4^+^ T-cell recovery may be observed in different immunological scenarios, suggesting that different causes could be behind a poor response to ART.

## Discussion

Few reports have attempted a comprehensive analysis of the contribution of CD8^+^ T-cell immune activation, immunological checkpoint molecules and CD4^+^ T-cell homeostasis to the pathogenesis of HIV infection [25–28]. In the present analysis, we took advantage of a large dataset of immunological data from HIV-infected ART-suppressed individuals to evaluate the goodness of different definitions of immune recovery by different machine learning and statistical approaches.

The lack of proper immune recovery in immunodiscordant individuals, defined by a poor CD4^+^ T-cell recovery, is a relevant clinical concern. Immunodiscordance is associated with adverse events occurring before ART initiation such as rapid progression [29] or late presentation [30], two phenomena that are relevant in developed and developing countries [31,32]. Furthermore, immunodiscordance determines the risk of clinical progression and death [9] and seems to be one of the main roadblocks to eliminate the differences in life expectancy between ART-treated and uninfected individuals [33].

Our results reveal that the CD4^+^ T-cell count 400 cells/μl cutoff segregates better immunological profiles of immunoconcordant and immunodiscordant individuals, highlighting differences principally in CD4^+^ T-cell death and activation between groups. In several studies, a cutoff of CD4^+^ T-cell count 200 cells/μl has been defined as a critical threshold for the risk of AIDS events or death [34–36]. However, a recent analysis has shown that risk of death or AIDS events follow a CD4^+^ T-cell count gradient in ART-treated participants, with clear risk for counts below 200 cells/μl but still some benefit for those individuals with a CD4^+^ T-cell count at least 500 cells/μl [9]. The persistent elevated levels of immune activation and death of CD4^+^ T cells in participants with lower CD4^+^ T-cell counts might explain this increased risk. In our analysis, the segregation of immunoconcordant and immunodiscordant individuals is better achieved by cutoff definitions rather than by increases in CD4^+^ T cells. This is probably associated with the structure of our cohort in which participants starting therapy a relatively high CD4^+^ T-cell count may show low cell increase, despite showing a fully immunoconcordant phenotype [17]. Adjusting for nadir CD4^+^ T-cell counts, or including a low nadir count as inclusion criteria would make both definitions (cutoff and increase) much more similar.

Our data also underscore the previously unnoticed existence of immunodiscordant participants with different immunological pattern. In fact, our data indicate that only a subgroup of immunodiscordant individuals (D-I, 31% of all immunodiscordant) fulfill all the previously defined features of these participants, that is low RTE frequency, high activation/cell death and high frequency and turnover of Treg cells [19,37–42]. On the other hand, the larger group of immunodiscordant individuals (D-II) showed low RTE frequency and a more modest contribution of activation and PD-1 expression. Finally, 22% of all immunodiscordant participants (D-III) showed an immunological profile closer to immunological full responders, with low activation, PD-1 expression, cell death and surprisingly a high frequency of naïve cells. We have ruled out a major role of HCV coinfection and cirrhosis in this phenotype. However, this group shows the lowest number of circulating CD8^+^ T cells, platelets, hematies, leukocytes and lymphocytes (Table 2 and Supplementary Fig. 4), suggesting that this particular phenotype could be the result of genetic or environmental factors altering lymphocyte distribution in the body rather than HIV-driven [43]. Moreover, lymphopenic status has been also observed in HIV-uninfected individuals [44]. Despite these immunological differences, all immunodiscordant groups showed similar evolution of CD4^+^ T-cell counts over time. A major limitation of our study is that the number of individuals analyzed is limited for subgrouping analyses, and this fact impedes to assess the impact of immunological profile on clinical events (AIDS related or not). However, the well described association of immunological alterations and CD4^+^ T-cell counts with clinical evolution [8,25] might suggest a better clinical evolution of Group III individuals, which will require further confirmation. An additional limitation of this study is the cross-sectional nature of the supervised/unsupervised analyses that impedes a proper longitudinal analysis from baseline (pre-ART) to address predictors of CD4^+^ T-cell recovery and early CD4^+^ T-cell redistribution events.

Classifying immunodiscordance could be particularly relevant in clinical settings, as distinct clinical approaches could be needed to reverse immunodiscordance in these subgroups. Noteworthy, such subclassification can be easily achieved with only three parameters (CD4^+^CD45RA^+^, CD4^+^HLA-DR^+^ and CD4^+^PD-1^+^ cells) with an overall 81% accuracy. ART intensification or immunomodulating approaches have been already tested to improve CD4^+^ T-cell recovery with limited effects [12–15,45–47]. A better participant selection and interventions more focused on specific immune alterations may help to succeed in this challenge. For instance, our data suggest that interventions aimed at improving thymic output may be useful in Group D-II but would be insufficient for Group D-I and may be unavailing in Group D-III.

In addition to classifying immunodiscordant participants, our approach also identified two subgroups of immunoconcordant individuals. Interestingly, main differences among groups were RTE frequency, activation and cell-death levels in both CD4^+^ and CD8^+^ T cells. Lower frequencies of CD4^+^ naive cells were accompanied by higher activation, PD-1 expression and death of CD4^+^ T cells and also by higher activation of CD8^+^ T cells. Potential explanations for this clustered phenotypes could be related to viral tropism, as X4 viruses are more tropic for naïve cells and may cause preferential depletion of these cells [48], or to the existence of residual viral replication in some individuals, that may drive both CD4^+^ and CD8^+^ T-cell activation [49]. Further characterization of individuals in subgroup C-II will provide the clues to explain suboptimal recovery of immune parameters and may contribute to define clinical strategies to improve them, such as ART intensification, drug switch or complementation with anti-inflammatory drugs.

In conclusion, our data show that complementing absolute cell count follow-up with measures of naive T-cell production and immune activation (in both CD4^+^ and CD8^+^ T cells) may help to segregate participants, especially those with immunodiscordant responses to optimize treatments aimed to increase their CD4^+^ T-cell counts. However, our data also suggest that the quality of CD4^+^ T cells is relevant. Therefore, increasing CD4^+^ T-cell counts may not be sufficient, in the absence of functional improvement, to impact clinical output as shown for IL-2 treatment [50].

## Acknowledgements

We are grateful to all the study participants for their time and participation in this study. This work was supported by the HIVACAT Program and the Spanish AIDS network ‘Red Temática Cooperativa de Investigación en SIDA RD12/0017/0002 project’ as part of the Plan Nacional R + D + I and cofunded by ISCIII-Subdirección General de Evaluación y el Fondo Europeo de Desarrollo Regional (FEDER) and by amfAR grant 109316 with support from FAIR. J.B. and C.C. are researchers from Fundació Institut de Recerca en Ciències de la Salut Germans Trias i Pujol supported by the ISCIII and the Health Department of the Catalan Government (Generalitat de Catalunya). M.M. is supported by BP-DGR AGAUR Postdoctoral Fellowship.

Author's contributions: M.M., J.C. and C.C. designed and performed immunophenotyping, cell death and ELISA assays. J.P.S., D.O., V.U. and J.V.F. performed statistical analyses. E.N., J.P. and B.C. recruited participants and collected clinical data. M.M., R.P., E.N., B.C. and J.B. interpreted the data. J.P.S., M.M. and J.B. coordinated the work and wrote the manuscript. All authors read and approved the final version of the manuscript.

### Conflicts of interest

There are no conflicts of interest.

## Supplementary Material

Supplemental Digital Content

## Figures and Tables

**Table 1:** Participant characteristics from the entire cohort (*n* = 196) and the subgroup of participants (*n* = 50) with additional immunological data.

	All participants (*n* = 196)	CD4^+^ T-cell count strata from all participants						K–W test or Chi sq test	Subgroup (*n* = 50)
		<200 (*n* = 19)	201–400 (*n* = 70)	401–600 (*n* = 44)	601–800 (*n* = 38)	801–1000 (*n* = 15)	>1001 (*n* = 10)		
Age (years)	45 (42–51)	48 (45–51)	48 (44–53)	45 (40–51)	44 (41–48)	45 (41–49)	44 (38–45)	0.02	45 (39–50)
Man, *n* (%)	150 (76)	16 (84)	58 (83)	33 (75)	24 (63)	10 (67)	9 (90)	*ns*	43 (86)
Time since HIV diagnosis (years)	13 (8–17)	14 (6–19)	13 (8–10)	13 (10–15)	12 (7–15)	14 (11–17)	11 (7–16)	*ns*	12 (6–18)
Time on ART (years)	11 (7–14)	8 (4–11)	10 (6–15)	11 (9–13)	11 (7–12)	11 (9–13)	11 (7–13)	*ns*	10 (4–18)
Nadir CD4^+^ cell count (cells/μl)	148 (65–272)	47 (22–85)	108 (42–159)	195 (81–273)	274 (162–320)	279 (249–406)	319 (172–496)	*<0.0001*	133 (23–274)
Nadir CD4^+^ cell count <200 cells/μl, *n* (%)	121 (61)	19 (100)	58 (83)	22 (50)	15 (39)	3 (20)	4 (40)	*<0.0001*	31 (62)
CD4^+^ T cells (% of lymphocytes)	26 (19–33)	14 (10–21)	19 (16–23)	27 (24–30)	33 (27–40)	38 (33–41)	40 (36–44)	*<0.0001*	25 (15–35)
CD4^+^ T-cell count (cells/μl)	444 (284–683)	162 (142–185)	289 (244–330)	486 (459–531)	700 (669–754)	912 (880–955)	1127 (1086–1541)	*<0.0001*	540 (233–865)
CD8^+^ T cells (% of lymphocytes)	46 (38–53)	55 (50–64)	51 (44–56)	47 (38–51)	39 (34–46)	36 (33–40)	37 (36–46)	*<0.0001*	47 (36–56)
CD8^+^ T-cell count (cells/μl)	800 (589–1067)	521 (286–767)	730 (610–974)	832 (642–1074)	802 (649–1114)	855 (739–1150)	1246 (971–1692)	*0.0002*	940 (764–1243)
Ratio CD4/CD8	0.54 (0.35-.087)	0.3 (0.2–0.4)	0.4 (0.3–0.5)	0.6 (0.5–0.8)	0.9 (0.6–1.1)	1.1 (0.8–1.2)	1.1 (0.8–1.20	*<0.0001*	0.53 (0.27–0.95)
Current ART, *n* (%)									
PI	89 (45)	12 (63)	35 (50)	18 (41)	16 (42)	5 (33)	3 (30)	*ns*	25 (50)
NNRTI	107 (54)	7 (37)	35 (50)	26 (59)	21 (55)	10 (67)	7 (70)	*ns*	24 (48)
HCV coinfection, *n* (%)	71 (36)	7 (37)	29 (41)	14 (32)	14 (37)	6 (40)	1 (10)	*ns*	12 (24)
HBV coinfection, *n* (%)	9 (5)	1 (5)	3 (4)	3 (7)	1 (2)	0 (0)	1 (10)	*ns*	3 (6)

Values (unless indicated) are given as Median (IQR); ART, antiretroviral therapy; HBV, hepatitis B virus; HCV, hepatitis C virus; NNRTI, non-nucleoside reverse transcriptase inhibitors; PI, protease inhibitor. K–W test: Kruskal–Wallis test for multiple comparisons. Chi sq test: chi square test.

**Table 2:** Main characteristics of subgroups identified.

	Discordant			Concordant		K–W test or Chi sq test	D-I vs D-II	D-I vs D-III	D-II vs D-III	C-II vs C-III	D-II vs C-II	D-III vs C-III
	D-I (*n* = 27)	D-II (*n* = 40)	D-III (*n* = 19)	C-II (*n* = 20)	C-III (*n* = 85)							
Age (years)	47 (42–52)	49 (45–54)	48 (46–50)	44 (39–54)	45 (41–48)	*0.001*						
Man, *n* (%)	27 (100)	32 (80)	12 (63)	19 (95)	55 (65)	*0.0004*						^*^
Years since HIV diagnosis	14 (5–20)	12 (8–19)	13 (8–19)	12 (8–15)	13 (10–16)	*ns*						
Time on ART (years)	8 (4–14)	10 (5–13)	10 (8–14)	11 (8–12)	11 (7–14)	*ns*						
Nadir CD4^+^ count (cells/μl)	90 (28–126)	88 (38–150)	139 (76–202)	163 (74–272)	258 (150–345)	*<0.0001*						^*^
CD4^+^ T-cell increase (cells/μl)	168 (92–198)	165 (82–216)	145 (99–222)	344 (254–556)	429 (330–572)	*<0.0001*					^***^	^***^
CD4^+^ T cells (%)	17 (12–20)	19 (15–21)	25 -20–32)	26 (24–31)	33 (27–39)	*<0.0001*						
CD4^+^ T-cell count (cells/μl)	249 (169–321)	263 (207–311)	290 (250–331)	545 (459–650)	683 (519–801)	*<0.0001*					^***^	^***^
CD8^+^ T cells (%)	55 (51–61)	50 (45–56)	46 (40–54)	45 (37–50)	39 (34–47)	*<0.0001*						
CD8^+^ T-cell count (cells/μl)	939 (669–1075)	697 (617–844)	510 (439–54)	871 (615–1288)	829 (719–1092)	*0.00*		^**^				^***^
Ratio CD4/CD8	0.3 (0.2–0.4)	0.4 (0.3–0.4)	0.5 (0,4–0.8)	0.6 (0.5–0.9)	0.8 (0.6–1.1)	*<0.0001*		^**^	^*^		^**^	^*^
Lymphocyte counts (cells/μl)	1600 (1350–1900)	1400 (1200–1700)	1200 (1100–1350)	2100 (1900–2475)	2200 (1800–2500)	*<0.0001*					^**^	^***^
PI-based ART, *n* (%)	13 (48)	22 (55)	9 (47)	9 (45)	32 (38)							
HCV coinfection (%)	14 (52)	15 (38)	6 (32)	6 (30)	29 (34)	*ns*						
HBV coinfection (%)	2 (7)	1 (2.5)	1 (5)	1 (5)	4 (5)	*ns*						
T-cell death				‘								
CD4^+^ T-cells death (%)	11.1 (8.1–16.0)	8.8 (6.9–11.5)	5.8 (4.2–9.8)	7.2 (5.0–11.3)	4.5 (3.6–5.7)	*<0.0001*						
CD8^+^ T-cells death (%)	6.9 (4.5–11.0)	7.8 (5.7–10.3)	6.0 (4.7–9.2)	11.8 (6.2–18.2)	6.1 (4.1–8.5)	*0.00*		^**^		^***^		^*^
Naive cells										^***^		
CD45RA^+^ (% of CD4^+^)	13.9 (9.4–30.7)	12.7 (9.5–22.3)	33.3 (26–42)	18.7 (11.1–23.1)	31 (24.0–38.3)	*<0.0001*						
CD45RA^+^CD31^+^ (% of CD4^+^)	7.1 (2.8–16.7)	7.6 (4.7–16.1)	20.0 (11.8–26.0)	9.9 (3.9–18.8)	20.8 (14.2–26.2)	*<0.0001*						
T-cell activation								^***^	^***^	^***^		
HLA-DR^+^ (% of CD4^+^)	36.1 (27.7–47.2)	11.9 (8.9–15.5)	8.5 (6.2–10.8)	12.7 (9.5–17.0)	6.4 (5.0–7.7)	*<0.0001*		^*^	^**^	^***^		
PD-1^+^ (% of CD4^+^)	14.6 (7.6–21.6)	9.5 (7.7–13.4)	8.3 (4.4–9.7)	11.2 (8.4–12.8)	6.5 (4.1–9.2)	*<0.0001*						
FAS^+^HLA-DR^+^ (% of CD4^+^)	20.1 (15.7–24.4)	9.9 (7.1–12.4)	6.8 (5.5–9.0)	8.3 (7.2–11.5)	4.8 (4.0–6.10)	*<0.0001*						
HLA-DR^+^ (% of CD8^+^)	32.4 (22.0–44.4)	19.9 (9.3–29.3)	12.7 (6.3–16.4)	25.1 (15.8–32.6)	14 (7.5–19.2)	*<0.0001*	^***^	^***^		^***^		
CD38^+^ (% of CD45RA^−^CD8^+^)	29.2 (20.0–48.7)	21.6 (14.3–35.9)	27.4 (13.6–38.7)	26.9 (18.2–49.2)	18.7 (11.3–27.9)	*0.00*	^**^	^***^		^***^		

Values (unless indicated) are given as median (IQR); ART, antiretroviral therapy; HBV, hepatitis B virus; HCV, hepatitis C virus; K–W test, Kruskal–Wallis test for multiple comparisons; Chi sq test, chi square test. For subgroup comparison:

^*^0.05 > *P* value > 0.01.

^**^0.01 > *P* value > 0.001.

^***^P value > 0.0001.
