# Supplementary material for: Antiretroviral therapy suppressed participants with low CD4+ T-cell counts segregate according to opposite immunological phenotypes
Source: AIDS. 2016 Sep 7;30(15):2275–87. doi: 10.1097/QAD.0000000000001205 (PMC5017266; doi:10.1097/QAD.0000000000001205)
Supplement: Supplemental Digital Content [file aids-30-2275-s001.pdf]

Supplementary Table 1: List of parameters used in this study. Left table shows parameters available for all subjects and right table shows parameters available for a smaller subset of subjects. Parameters are grouped by function.

Parameter	Unit
Clinical parameters	
1 Time since diagnosis	years
2 Time on ART	years
3 Treatment	PI or NNRTI
4 Drug	Kaletra
	Nevirapine
	ATV
	Efavirenz
	FosApv
	SQV
5 HBV infection	Yes or No
6 HCV infection	Yes or No
7 Detectable VL HCV	Yes or No
8 Age	Years
9 Gender	Male or Female
10 Nadir CD4 T cell count	cells/µl
11 Percentage of CD8	%
12 Absolute CD8 T cell counts	cells/µl
13 Ratio CD4/CD8	-
Microbial translocation	
14 Soluble CD14	ng/mL
T-cell cell death	
15 Total CD4 cell death (day 0)	% of CD4 T cells
16 Total CD8 cell death (day 0)	% of CD8 T cells
17 Total CD4 cell death (day 1)	% of CD4 T cells
18 CD4 T cell Necrosi (day 1)	% of CD4 T cells
19 CD4 T cell total apoptosis (day 1)	% of CD4 T cells
20 CD4 T cell intrinsic apoptosis (day 1)	% of CD4 T cells
21 CD4 T cell extrinsic apoptosis (day 1)	% of CD4 T cells
22 Total CD8 cell death (day 1)	% of CD8 T cells
23 CD8 T cell Necrosi (day 1)	% of CD8 T cells
24 CD8 T cell total apoptosis (day 1)	% of CD8 T cells
25 CD8 T cell intrinsic apoptosis (day 1)	% of CD8 T cells
26 CD8 T cell extrinsic apoptosis (day 1)	% of CD8 T cells
CD4 T cells	
27 CD45RA^{-}CD31^{+}	% of CD4 T cells
28 CD45RA^{+}CD31^{+}	% of CD4 T cells
29 CD45RA^{+}CD31^{-}	% of CD4 T cells
30 CD45RA^{-}CD31^{-}	% of CD4 T cells
31 CD45RA^{-}CD38^{+}	% of CD4 T cells
32 CD45RA^{+}CD38^{+}	% of CD4 T cells
33 CD45RA^{+}CD38^{-}	% of CD4 T cells
34 CD45RA^{-}CD38^{-}	% of CD4 T cells
35 CD45RA^{+}	% of CD4 T cells
36 CD45RA^{-}	% of CD4 T cells
37 CD38^{+}	% of of CD45RA– CD4 T cells
38 CD31^{+}	% of of CD45RA+ CD4 T cells
39 FAS^{+}PD-1^{-}	% of CD4 T cells
40 FAS^{+}PD-1^{+}	% of CD4 T cells
41 FAS^{-}PD-1^{+}	% of CD4 T cells
42 FAS^{-}PD-1^{-}	% of CD4 T cells
43 FAS^{+}HLA-DR^{-}	% of CD4 T cells
44 FAS^{+}HLA-DR^{+}	% of CD4 T cells
45 FAS^{-}HLA-DR^{+}	% of CD4 T cells
46 FAS^{-}HLA-DR^{-}	% of CD4 T cells
47 PD-1+HLA-DR^{-}	% of CD4 T cells
48 PD-1+HLA-DR^{+}	% of CD4 T cells
49 PD-1^{-}HLA-DR^{+}	% of CD4 T cells
50 PD-1^{-}HLA-DR^{-}	% of CD4 T cells
51 HLA-DR^{+}	% of CD4 T cells
52 PD-1+	% of CD4 T cells
53 FAS^{+}	% of CD4 T cells
CD8 T cells	
54 CD45RA^{-}CD31^{+}	% of CD8 T cells
55 CD45RA^{+}CD31^{+}	% of CD8 T cells
56 CD45RA^{+}CD31^{-}	% of CD8 T cells
57 CD45RA^{-}CD31^{-}	% of CD8 T cells
58 CD45RA^{-}CD38^{+}	% of CD8 T cells
59 CD45RA^{+}CD38^{+}	% of CD8 T cells
60 CD45RA^{+}CD38^{-}	% of CD8 T cells
61 CD45RA^{-}CD38^{-}	% of CD8 T cells
62 CD45RA^{+}	% of CD8 T cells
63 CD45RA^{-}	% of CD8 T cells
64 CD38^{+}	% of of CD45RA– CD8 T cells
65 CD31^{+}	% of of CD45RA+ CD8 T cells
66 FAS^{+}HLA-DR^{-}	% of CD8 T cells
67 FAS^{+}HLA-DR^{+}	% of CD8 T cells
68 FAS^{-}HLA-DR^{+}	% of CD8 T cells
69 FAS^{-}HLA-DR^{-}	% of CD8 T cells
70 HLA-DR^{+}	% of CD8 T cells
71 FAS^{+}	% of CD8 T cells
CMV serology	
72 BIOFLASH CMV IgG	AU/mL
73 BIO-FLASH CMV IgM	AU/mL
74 BIOELISA CMV	OD-COV

Parameter	Unit
CD4 T cells	
75 Naïve	% of CD4 T cells
76 Central memory	% of CD4 T cells
77 Transitional memory	% of CD4 T cells
78 Effector	% of CD4 T cells
79 CD27^{-}CD45RA^{-}CCR7^{+}	% of CD4 T cells
80 CD27^{-}CD45RA^{+}CCR7^{+}	% of CD4 T cells
81 CD27^{-}CD45RA^{-}CCR7^{-}	% of CD4 T cells
82 Effector memory	% of CD4 T cells
83 TEMRA	% of CD4 T cells
84 CD57^{+}	% of CD4 T cells
85 CD57^{+}	% of naïve CD4 T cells
86 CD57^{+}	% of central memory CD4 T cells
87 CD57^{+}	% of transitional memory CD4 T cells
88 CD57^{+}	% of effector memory CD4 T cells
89 Naïve	% of CD8 T cells
90 Central memory	% of CD8 T cells
91 Transitional memory	% of CD8 T cells
92 Effector	% of CD8 T cells
93 CD27^{-}CD45RA^{-}CCR7^{+}	% of CD8 T cells
94 CD27^{-}CD45RA^{+}CCR7^{+}	% of CD8 T cells
95 CD27^{-}CD45RA^{-}CCR7^{-}	% of CD8 T cells
96 CD27^{-}CD45RA+CCR7^{-}	% of CD8 T cells
97 Revertant	% of CD8 T cells
98 CD57^{+}	% of CD8 T cells
99 CD57^{+}	% of naïve CD8 T cells
100 CD57^{+}	% of central memory CD8 T cells
101 CD57^{+}	% of transitional memory CD8 T cells
102 CD57^{+}	% of effector memory CD8 T cells
103 CD57^{+}	% of revertant CD8 T cells
104 Ki67^{+}	% of CD4 T cells
105 Ki67^{+}	% of CD4 non-Treg cells
106 Treg classic (CD25^{hi}FOXP3^{+})	% of CD4 T cells
107 Ki67^{+}	% of CD4 Treg classic
108 Treg Natural	% of CD4 T cells
109 Treg Effector	% of CD4 Treg natural
110 Treg CD25^{hi}CD127^{low}	% of CD4 T cells
111 Treg CD25^{bright}	% of CD4 T cells
112 Ki67^{+}	% of CD4 Treg CD25^{bright}
113 Treg CD25^{low}	% of CD4 T cells
114 Ki67^{+}	% of CD4 Treg CD25^{low}
115 Treg CD25^{Bright}FOXP3^{Bright}	% of CD4 T cells
116 Ki67^{+}	% of CD4 Treg CD25^{bright}FOXP3^{bright}
117 Treg CD25^{low}FOXP3^{low}	% of CD4 T cells
118 Ki67^{+}	% of CD4 Treg CD25^{low}FOXP3^{low}
119 Ki67^{+}	% of CD8 T cells
120 Ki67^{+}	% of CD8 non-Treg cells
121 Treg classic (CD25^{hi}FOXP3^{+})	% of CD8 T cells
122 Ki67^{+}	% of CD8 Treg classic

Supplementary Figure 1

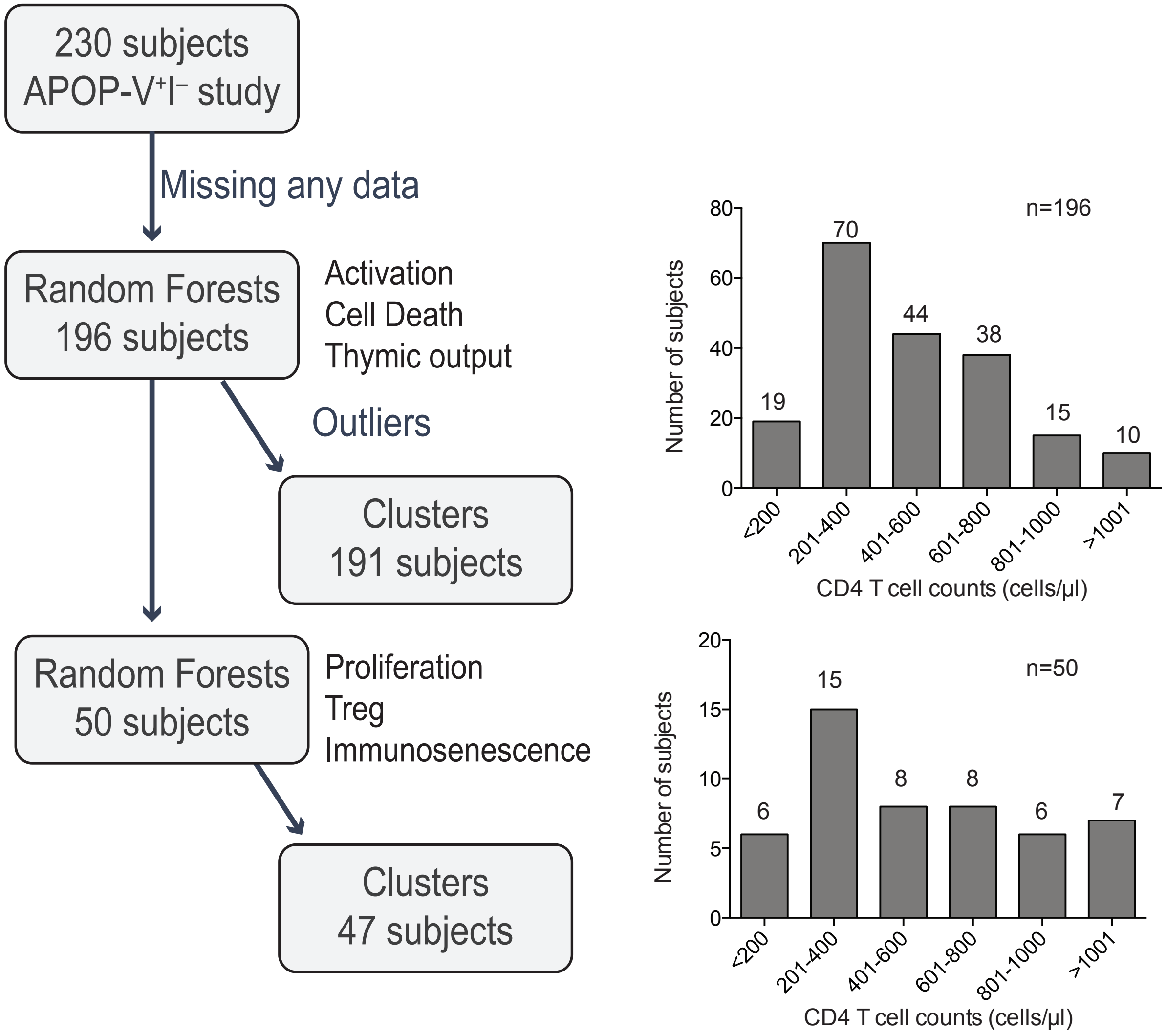

Supplementary Figure 1. Analysis strategy. The figure shows the starting cohort (references 19 and 20). Random forest approach classification was performed with 196 individuals with a complete dataset (see supplementary Table 1). The distribution of the different CD4 T-cell counts strata is shown for this subset. Hierarchical clustering and heatmaps were analyzed in a subgroup of 191 subjects after removal of outliers. In a subgroup of subjects, additional Treg, T-cell differentiation and proliferation parameters were analyzed (n=50). Strata according to CD4 T-cell counts is also shown for this subset.

Supplementary Figure 2

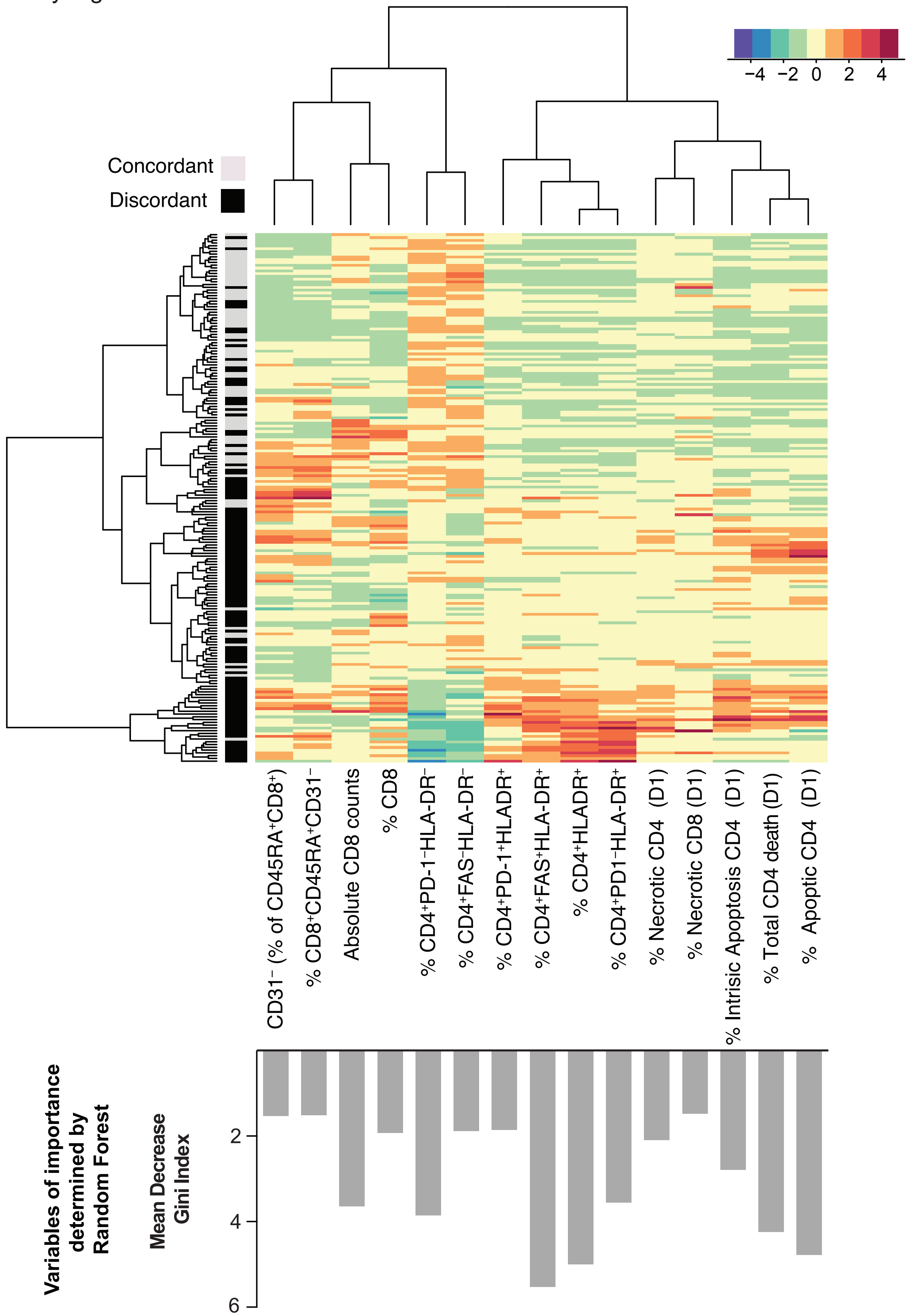

Supplementary Figure 2. Clustering of subjects according to variables of importance of the ΔCD4 T-cell count value 350 cells/μL. Heatmap of 191 individuals generated using relevant parameters for classification shown in Figure 2. The output heatmap shows one small group of mostly immunodiscordant individuals (Group I) and a major group of intermingled immunodiscordant and immunoconcordant individuals. Color key is shown in the upper right side and Gini index for each variable is shown in the lower histogram.

Supplementary Figure 3

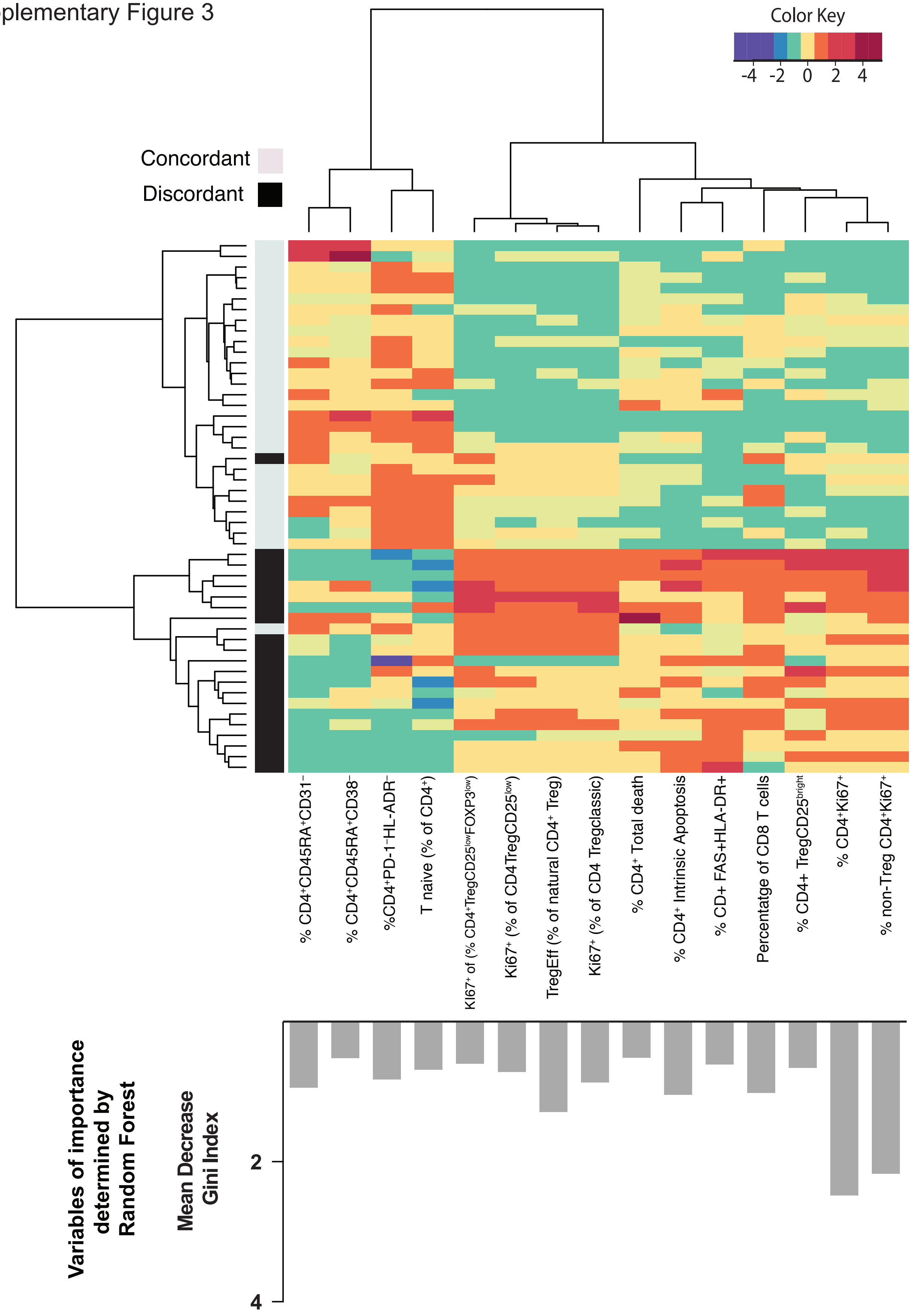

Supplementary Figure 3. Clustering of subjects according to variables of importance obtained from Treg, proliferation and differentiation data for a CD4 T-cell count cutoff 400 cells/µL. Heatmap of 49 individuals generated from activation, cell death, thymic output, Treg, proliferation, differentiation and immunosenescence data shows a clear segregation of immunodiscordant and immunoconcordant individuals. Color key is shown in the upper right side and Gini index for each variable is shown in the lower histogram.

Supplementary Figure 4

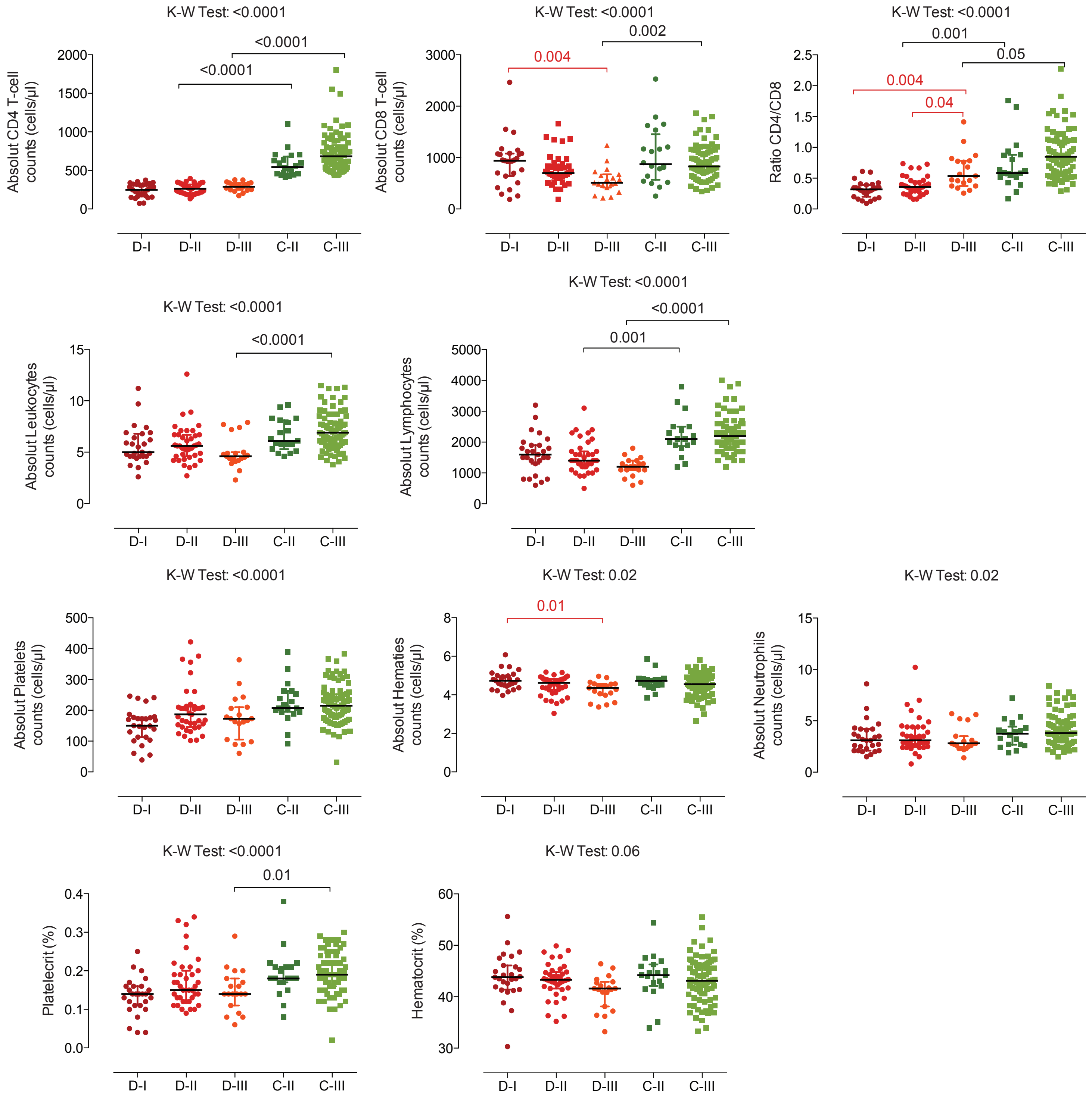

Supplementary Figure 5

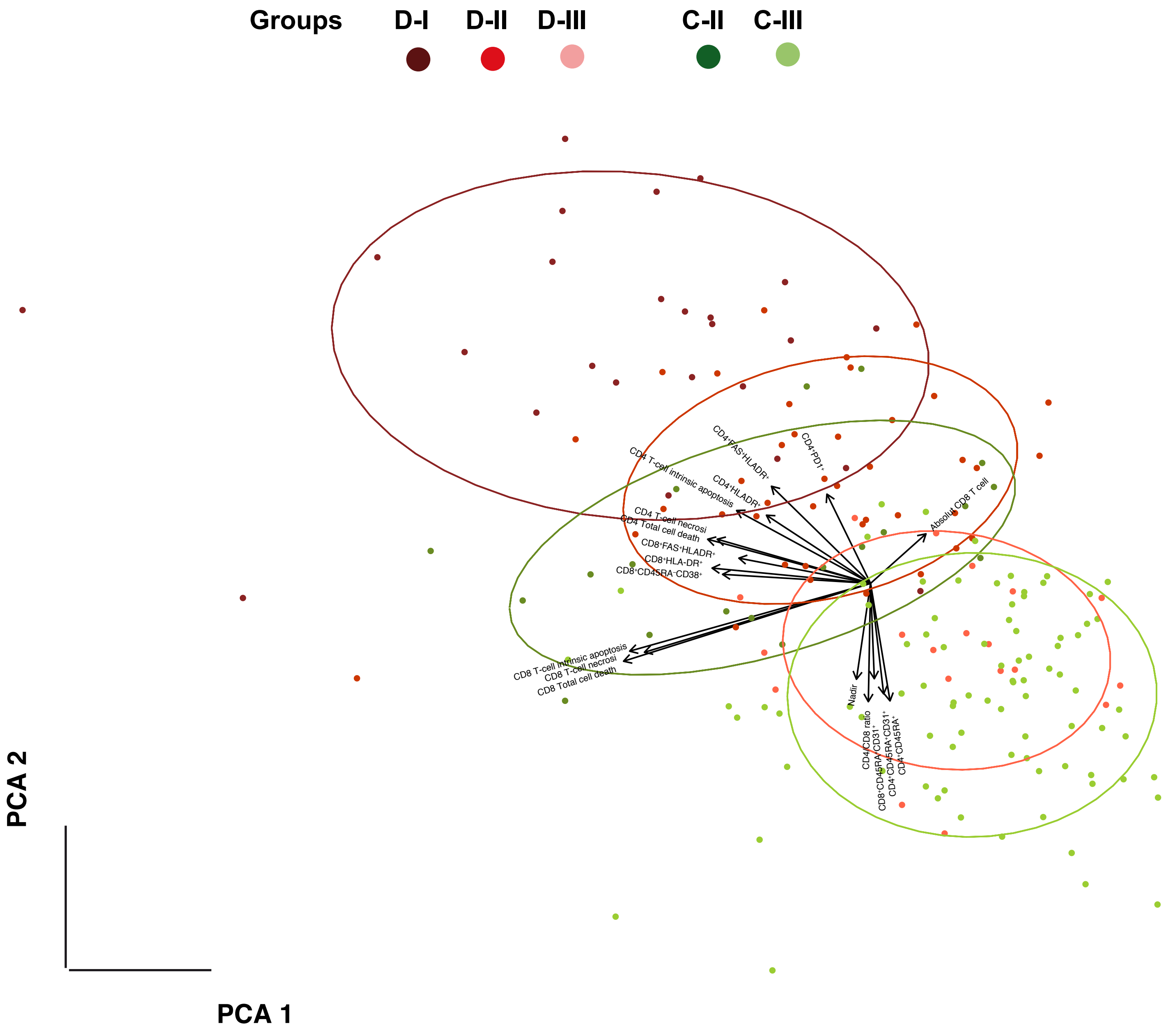

Supplementary Figure 4. PCA analysis of thymic output, cell death and activation data in CD4 and CD8 T cells. Data were standardized and a varimax rotation was performed on the two first components. Before rotation, the first two components accounted for 48% of the overall variability. Ellipses for each group represent a multivariate Normal contour at the 68% of confidence level (corresponding to a distance of one standard deviation from the mean). Colors represent the groups of previous clustering results

Supplementary Figure 6

A

	D-I	D-II	D-III	Classification error
**D-I**	**26**	1	0	0.037
**D-II**	3	**29**	8	0.275
**D-III**	0	4	**15**	0.210

B

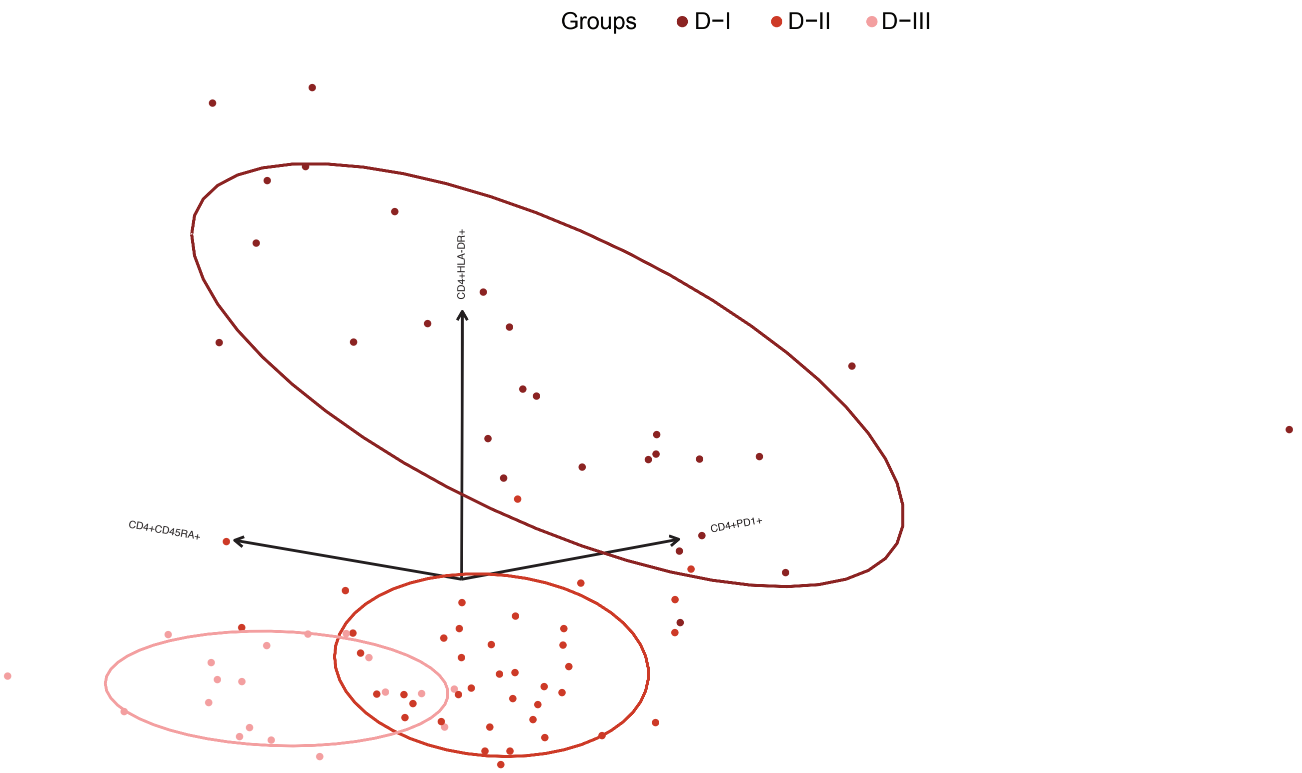

Supplementary Figure 5. Simplified classification of immunodiscordant subjects. The following parameters: frequency of $CD4^{+}CD45RA^{+}$, $CD4^{+}HLA\text{-}DR^{+}$ and $CD4^{+}PD\text{-}1^{+}$ cells were selected according to differences among immunodiscordant subgroups in order to establish a simplified classification algorithm. (A). Confusion matrix to assess the ability of this simple classification algorithm to segregate D-I, D-II and D-III individuals. (B). PCA analysis of this classification. Ellipses for each group represent a multivariate Normal contour at the 68% of confidence level (corresponding to a distance of one standard deviation from the mean). Colors represent the groups of previous clustering results
